# Self-Compassion as a Key Factor of Subjective Happiness and Psychological Well-Being among Greek Adults during COVID-19 Lockdowns

**DOI:** 10.3390/ijerph20156464

**Published:** 2023-07-27

**Authors:** Kyriaki Sotiropoulou, Christina Patitsa, Venetia Giannakouli, Michail Galanakis, Christiana Koundourou, Georgios Tsitsas

**Affiliations:** 1 Department of Psychology, School of Health Sciences, Neapolis University Pafos, 8042 Pafos, Cyprus; 2 Department of Archival, Library and Information Studies, University of West Attica, 122 43 Athens, Greece; 3 Department of Nutrition and Dietetics, School of Health Science and Education, Harokopio University, 176 76 Athens, Greece

**Keywords:** self-compassion, psychological well-being, subjective happiness, resilience, meaning in life, COVID-19

## Abstract

The present study examined the association and complementary effect of self-compassion on the subjective happiness and psychological well-being of adults during the COVID-19 pandemic. The study was based on a concurrent correlational design to examine relationships between self-compassion, subjective happiness, psychological well-being, resilience, and the meaning in life. Data were collected via a battery of questionnaires and analyzed, focusing on the above variables. The sample of this study (N = 526) consisted of Greek professionals in education and university students. The results showed that there is a strong positive relationship between self-compassion and subjective happiness, and between self-compassion and psychological well-being. The findings suggest that an attitude of self-compassion may well influence the development of psychological well-being and increase the subjective happiness of adults during the distressing era of a long-term pandemic. The results also indicated a positive relationship between self-compassion and meaning in life and showed that self-compassion is a prerequisite for resilience, which in turn may serve as a moderator of psychological well-being and subjective happiness.

## 1. Introduction

The development and promotion of health services, and long-term psychological services in particular, have become top priority worldwide, following the spread of the COVID-19 pandemic [1,2,3,4]. The spread of COVID-19 as a life-threatening situation has changed our daily lives in an unprecedented way, which has reasonably taken a toll on people’s mental health, and consequently fueled scientific discussion about finding ways to save, maintain or even improve individuals’ well-being and feelings of happiness, and has placed people on a quest to find the best possible way to provide practical and emotional support. In this context, in the last two years, many researchers around the world have tried to explore skills, traits and habits that could make a difference to the lives and feelings of adults facing the pandemic [5]. Many scholars have indicated the significant role of three psychological parameters—self-compassion (SC), psychological well-being (PWB) and subjective happiness (SH)—as catalysts in coping with the pandemic and new life circumstances [6,7,8]. These circumstances include stressful lockdowns, a reduced income, homeschooling and working from home, severe changes in social interaction and an increase in feelings of anxiety [9]. Nevertheless, it is important to note that the COVID-19 pandemic is itself a highly stressful situation, whetherassociated with a lockdown and affects adult mental health in a variety of ways.

Psychological well-being (PWB) and changes in adult mental health during the pandemic remain one of the most important concerns of governments at a global level. PWB refers to all kinds of positive interaction with oneself and others, as well as positive attitudes toward it; it means being in control of oneself and having a sense of personal development. As for its components, subjective well-being (SWB) is considered under the ancient Greek concepts of hedonism and eudaimonia, that is hedonic and eudemonic well-being [10,11], which means being satisfied, happy and psychologically functional at the same time. Following the ancient terms, hedonic well-being is used to describe the personal aspect and subjective evaluation of feeling happy and satisfied with one’s life status (subjective well-being—SWB), while eudemonic well-being refers to a more psychological functional state (psychological well-being—PWB).

In addition, there is scientific evidence that subjective happiness may influence the quality of life during the pandemic [12]. The concept of subjective happiness (SH) is understood as a broader term for a person’s well-being, which is an indicator of how happy or unhappy one feels, not only as a personal assessment, but also in comparison with others [13,14].

Recent research has also shown that self-compassion can moderate the negative effects of COVID-19 on adult mental health [6]. Self-compassion means finding a way to ease oneself into difficult situations and to be kind and understanding about one’s own weaknesses. Self-compassion has its origins in the spiritual culture of Buddhism, which is about respecting oneself and others, being kind, calm, empathetic, and patient when dealing with problems and difficult times, and supporting oneself and being strong in times of distress and disaster [15]. This trait—which mainly characterizes mentally healthy people—may even protect them from increasing psychopathological symptoms, as it activates resilience mechanisms against stress. That is, people with self-compassion are more adaptive and flexible, and manage to regulate their own negative emotions and the difficulties of everyday life, thus promoting their mental health and protecting them from dysfunctional thoughts that promote psychopathology. Even in older adults, traits of self-compassion appear to help them cope better with stressors of poor health, making them more resilient and develop psychological well-being. That is, elderly people who are highly stressed about their health problems and have not developed mechanisms of self-compassion are less resilient and more likely to lead unhappy lives [16].

Research has shown that self-compassion is associated with psychological well-being before and during the recent pandemic. Many studies that have investigated the relationship between these two variables have emphasized the positive relationship between them in recent decades [17,18,19]. The relationship between self-compassion and subjective well-being is also evident during the COVID-19 pandemic [20,21,22]. According to the study by Nguyen and et al. [23], self-compassion is a key factor in psychological well-being during the pandemic because it provides people with a sense of safety and security and helps them fight against great negative feelings. Moreover, self-compassion not only has a strong effect on psychological well-being, but also proves to be a very effective weapon against the anxiety caused by the virus, thus mitigating the negative psychological consequences.

Scientific evidence suggests that self-compassion is also positively correlated with happiness and optimism, both states indicative of a mentally healthy adult. A previous study by Lyubomirsky and Lepper [13] indicated that self-compassion is directly related to subjective happiness, especially when considering positive relationships with others during difficult times, as people tend to compare their lives with others during such times. Similar findings [24,25], demonstrated the direct effect of self-compassion on psychological well-being and qualities such as optimism, happiness, and reduction in stress levels. Similarly, the relationship between self-compassion and subjective happiness was also confirmed during the COVID-19 pandemic [6].

As the strange and stressful times of the pandemic continue, most research focuses on characteristics and qualities related to distress and negative emotions. What does not seem to have been truly explored is the relationship between self-compassion and well-being during the difficult times of ongoing lockdowns and restrictive measures. The present study therefore aims to investigate the related and complementary effects of self-compassion on adults’ subjective happiness and psychological well-being during the COVID-19 pandemic. In particular, research directly addressing the relationship between self-compassion and subjective well-being among adults in higher education (students or workers) during the COVID-19 era has never been conducted in Greece and Cyprus, so researchers are interested in knowing this relationship.

In examining the multidimensional relationships between self-compassion, subjective happiness, and psychological well-being, other factors that have been shown to improve the quality of life during the pandemic, which should be considered from a holistic perspective, are resilience and meaning in life [26,27]. The purpose of the present study is to address these considerations. The concept of resilience refers to inner psychological qualities and skills that one cultivates to combat stress. Resilient individuals are characterized by inner strength and a positive outlook on life, while being flexible and effective in dealing with adversity in difficult situations [28]. Meaning in life means having a goal that makes sense to you, that connects you to yourself, and that provides you with a future perspective that you enjoy working for and are motivated by [29].

In this context, the following six research hypotheses, derived from the literature, have guided the present study:

**Hypothesis** **1:** *There is a positive relationship between self-compassion andresilience.*

**Hypothesis** **2.1:** *There is a positive relationship between self-compassion and subjective happiness.*

**Hypothesis** **2.2:** *There is a positive relationship between self-compassion and psychological well-being.*

**Hypothesis** **3:** *Resilience acts as a mediator between self-compassion and psychological well-being.*

**Hypothesis** **4:** *Subjective happiness acts as a mediator between self-compassion, resilience, meaning in life, and psychological well-being.*

**Hypothesis** **5:** *Self compassion directly increases subjective happiness.*

**Hypothesis** **6:** *Subjective happiness predicts psychological well-being and vice versa.*

## 2. Materials and Methods

### 2.1. Participants

The sample of this study (N = 526) consisted mainly of females (90.3%). A total of 42% of the sample were in the age group of 22–30 years, and 25% were 31–38 years old. The majority of the sample had a bachelor’s degree (65%), were single (53%), and were students (81%). Most participants were employed outside of tertiary education field (74%). Detailed results of participants’ demographic characteristics are presented in Table 1.

### 2.2. Procedure

The research design was based on a correlational field approach. Educational professionals and university students completed an online survey that included measures of self-compassion, psychological well-being, subjective happiness, and psychological resilience. The questionnaires were completed individually using Google forms. All participants received an informed consent form assuring confidentiality. A total of 66% of the total participants returned a completed questionnaire containing the variables of the study.

### 2.3. Measures

#### 2.3.1. Subjective Happiness Scale

The Greek version of the subjective happiness scale (SHS) was used to examine the subjectivity of individuals global happiness using four items rated on a 7-point Likert scale, with higher scores reflecting greater happiness [28]. Standardization of the subjective happiness scale (SHS) in a Greek sample proved satisfactory psychometric qualities for the Greek population [29].

#### 2.3.2. Presence of Meaning in Life Questionnaire

The meaning in life questionnaire (MLQ) was used to measure the presence of meaning (how much respondents perceive their lives to be meaningful) using five items rated on a 7-point Likert-type scale, ranging from 1 (absolutely true) to 7 (absolutely untrue) (e.g., “My life has a clear sense of purpose”). We used the Greek version of the instrument, which has demonstrated good internal consistency in a Greek sample [30].

#### 2.3.3. Connor–Davidson Resilience Scale

The Connor–Davidson resilience scale (CD-RISC) was used to measure individuals’ ability to cope with and recover from stress using 25 items rated on a 5-point Likert scale, with higher scores reflecting greater resilience (e.g., “Can handle unpleasant feelings”) [31]. We used the Greek version of the instrument, which has shown good internal consistency in a Greek sample [29].

#### 2.3.4. Self-Compassion Scale

Self-compassion was measured by the self-compassion scale (SCS) [32], in the Greek version [33,34], which consists of 26 items assessing six different aspects of self-compassion: self-kindness (e.g., “I try to be loving to myself when I feel emotional pain”), self-judgment (e.g., “I am intolerant and impatient with the aspects of my personality that I don’t like”), common humanity (“When I am down and out, I remind myself that there are many of other people in the world who feel the same way I do”), isolation (e.g., “When I fail at something that is important to me, I tend to feel alone with my failure”), mindfulness (“When something painful happens, I try to take a balanced view of the situation”), and overidentification (“When I feel down, I tend to obsess and fixate on everything that is wrong”). Each item was rated on a 5-point response scale, ranging from 1 (almost never) to 5 (almost always). Mean scores are then averaged (after reverse-coding negative items) to create an overall self-compassion score ranging from 26 to 130. Higher scores correspond to higher levels of self-compassion. Standardization of the Greek version of the SCS showed satisfactory reliability and validity, and the factorial structure of the scale was found to be consistent with the results of previous studies from other countries [32,33,34].

#### 2.3.5. Differential Emotions Scale-Modified (DESMOD)

We used the Greek version of the instrument the emotions scale-modified (DESMOD) instrument to assess a person’s emotions and psychological well-being [35]. The DESMOD asks participants to recall the past two weeks and rate their strongest experience of each of 20 specific emotions on a 5-point Likert scale (1—not at all to 5—extremely). The positive emotions subscale (PES) is a composite of nine positive emotions. The negative emotions subscale (NES) is a composite of seven negative emotions.

#### 2.3.6. Scale of Demographic Variables

For the purpose of the research, a scale was created by the research team to measure the demographic variables, including gender, age, educational status, marital status, place of residence, existence of health problems, and habits during quarantine.

## 3. Results

Data statistical analysis was performed with SPSS (version28) [36] and JAMOVI (version2.2.5) [37]. Demographic-related variables were described with absolute and relative frequencies (N, %). The reliability of each scale was examined using the Cronbach’s alpha index. Normality of the data was determined by examining the skewness/kurtosis values and histograms. Pearson’s correlation coefficient was used to test Hypotheses 1, 2.1, and 1.2. For Hypothesis 3, a simple mediation analysis was performed with resilience as the mediator, self-compassion as the IV, and psychological well-being as the DV. Hypothesis 4 was examined by developing a mediation model with subjective happiness as the mediator, psychological well-being as the DV, and resilience and self-compassion as the IVs. Then, hierarchical logistic regression models were produced for hypotheses 5 and 6. More specifically, to test whether self-compassion directly increases subjective happiness, a four-step model was developed in which self-compassion was included in the first step, meaning of life in the second step, resilience in the third step, and demographic dummies in the fourth step. For Hypothesis 6 and the prediction of psychological well-being through subjective happiness, a three-step model was examined, i.e., in the first step, only subjective happiness was inserted, then in the second step, self-compassion, meaning of life, and resilience, while in the third step, the model was adjusted to demographics. The same rationale was implemented for the prediction of subjective happiness by psychological well-being.

### 3.1. Reliability Analysis

The reliability results of the study instruments were satisfactory. They are presented in Table 2.

The subjective happiness scale in this study had a reliability index of α = 0.825.

The DESMOD scale for negative emotions (eight items) presented a reliability index of α = 0.811, and for positive emotions (nine items) had α = 0.882 in this study.

The Connor–Davidson resilience scale had a reliability index of α = 0.919.

The Self-compassion scale presented a reliability index of α = 0.925.

### 3.2. Correlations

According to the results of Table 3, there was a strong positive relationship between self-compassion and resilience (Pearson *r* = 0.610, *p* < 0.01), self-compassion and subjective happiness (Pearson *r* = 0.607, *p* < 0.01), and self-compassion and psychological well-being (Pearson *r* = 0.569, *p* < 0.01). The relationship between self-compassion and the meaning of life was positive with a medium effect size (Pearson *r* = 0.382, *p *< 0.01). Hence hypotheses 1, 2.1, and 2.2 are confirmed.

To investigate Hypothesis 3, a simple mediation analysis was performed. The outcome variable was psychological well-being, and the predictor variable was self-compassion. The mediator variable was resilience. The indirect, direct, and total effects, as well as path coefficients are presented in Table 4. Because zero is not in either the indirect or direct effects of the 95% confidence interval, these effects were significantly different from zero at *p* < 0.05 (two-tailed).

Therefore, resilience fully mediated the relationship between self-compassion and psychological well-being with a significant indirect effect (a × b = 0.008, 95%CI 0.005, 0.011) and direct effect (c = 0.023, 95%CI 0.019, 0.028). Participants who demonstrated high levels of self-compassion also demonstrated high levels of resilience and psychological well-being, while those who demonstrated high levels of resilience, demonstrated even higher levels of psychological well-being. The overall effect of self-compassion on psychological well-being was also significant. The total effect of self-compassion on psychological well-being was also significant (c′ = 0.032, 95%CI 0.028, 0.036).

**Figure 1:**
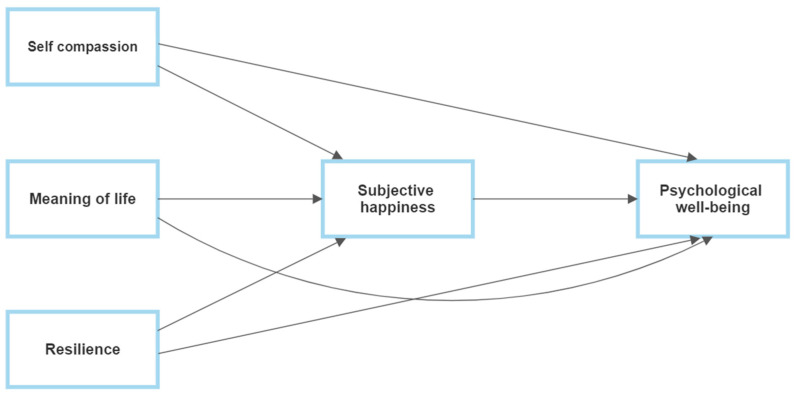
Model for mediation of subjective happiness in the relationship between self-compassion, meaning of life and resilience with psychological well-being.

To examine Hypothesis 4, a mediation analysis was conducted based on the model presented in Figure 1. The outcome variable was psychological well-being, and the predictor variables were self-compassion, meaning of life, and resilience. The mediator variable was subjective happiness.

Indirect, direct, and total effects, as well as path coefficients are shown in Table 5. For meaning of life, thereis neither a significant direct effect (c = −0.002, 95%CI −0.008, 0.008) nor a total effect (c′ = 0.004, 95%CI −0.004, 0.012). However, the indirect effect was significant (a × b = 0.004, 95%CI 0.005, 0.011), implying that participants who indicated high levels of meaning of life also exhibited high levels of subjective happiness, and they presented higher levels of psychological well-being through high levels of subjective happiness.

Moreover, subjective happiness fully mediated the relationship between self-compassion and psychological well-being with significant indirect (a × b = 0.009, 95%CI 0.001, 0.007) and direct effects (c = 0.015, 95%CI 0.009, 0.019), in a way that participants who indicated high levels of self-compassion also showed high levels of subjective happiness and psychological well-being, and through high levels of subjective happiness, presented higher levels of psychological well-being. The total effect of self-compassion on psychological well-being was also significant (c′ = 0.023, 95%CI 0.018, 0.029).

Also, subjective happiness fully mediated the relationship between resilience and psychological well-being with significant indirect (a × b = 0.008, 95%CI 0.005, 0.011) and direct effects (c = 0.007, 95%CI 0.001, 0.013), in a way that participants who indicated high levels of resiliencealso had high levels of subjective happiness and psychological well-being, and through high levels of subjective happiness, they presented higher levels of psychological well-being. The total effect of resilience on psychological well-being was also significant (c’ = 0.015, 95%CI 0.009, 0.022).

To examine the fifth hypothesis of the direct positive link between self-compassion and subjective happiness, hierarchical linear regression was performed with subjective happiness as the dependent variable and self-compassion as the main independent variable (Table 6). In the first step (model 1), only self-compassion was introduced in the model with a positive effect (β = 0.603, *p* < 0.001). Next, meaning in life was also introduced as a control variable (model 2) and self-compassion remained significant for subjective happiness with a reduced effect size (β = 0.522, *p* < 0.001). When resilience was also introduced (model 3), self-compassion remained significant for subjective happiness (β = 0.378, *p* < 0.001). In the final step, the model was adjusted to demographics (model 4). After accounting for all related variables and demographics, it was concluded that self-compassion has a direct positive effect on subjective happiness (β = 0.391, *p* < 0.001), thus confirming Hypothesis 5.

To test the last hypothesis, two analyses were performed. First, with subjective happiness as the predictor and psychological well-being as the outcome (Table 7), and then with psychological well-being as the predictor and subjective happiness as the outcome (Table 7). Hierarchical regression results in Table 7 show that in the first step (model 1), subjective happiness has a positive effect on psychological well-being (β = 0.625, *p* < 0.001). Next, controlling for self-compassion, the meaning of life and resilience (model 2), subjective happiness remained a significant predictor of psychological well-being with a reduced effect size (β = 0.413, *p* < 0.001). In the final step (model 3), the model was adjusted to demographics, and subjective happiness was found to have a direct positive effect on psychological well-being (β = 0.410, *p* < 0.001). The final model explains 45.6% of the variance in psychological well-being.

Moreover, hierarchical regression results in Table 8 for psychological well-being as a predictor of subjective happiness, show that in the first step (model 1), psychological well-being has a positive effect on subjective happiness (β = 0.625, *p* < 0.001). When controlling for self-compassion, the meaning of life, and resilience (model 2), psychological well-being remained a significant predictor of subjective happiness with a reduced effect size (β = 0.353, *p* < 0.001). In the final step (model 3), the model is adjusted to demographics, and it was concluded that psychological well-being has a direct positive effect on subjective happiness (β = 0.344, *p* < 0.001). The final model explains 54.3% of the variance in subjective happiness.

This is a more robust and parsimonious fit than psychological well-being, leading to subjective happiness. An explanation for this phenomenon may dependon the fact that self-compassion, resilience, and psychological well-being refer or depend on the relationship between the self and the environment, while at the same time, subjective happiness is more of an internal and individual construct regardless of one’ssurroundings.

## 4. Discussion

During the COVID-19 pandemic, well-being and positive psychology in general have become even more important. Research has shown the importance of finding individual ways to help oneself and cope with this life-threatening tsunami. With this in mind, this study sought to examine the role of a self-compassionate attitude in adults to help them increase their psychological well-being and sense of happiness during the difficult times of the pandemic.

According to the results of the study, the data showed that there were strong positive relationships between self-compassion and resilience, self-compassion and subjective happiness, and self-compassion and psychological well-being. Indeed, self-compassion has been found to be a protective shield against psychopathological symptoms as it activates resilience mechanisms against stress [38]. That is, people with self-compassion are more adaptive and flexible and are able to regulate their own negative emotions and hassles of everyday life, thereby promoting their mental health, protecting themselves from dysfunctional thoughts that promote psychopathology. Recent research studies have also confirmed the positive link between resilience and self-compassion, suggesting that both promote mental health and protect against stress during the pandemic period [39,40].

The correlation between self-compassion and subjective happiness is evidenced in the literature, where self-compassion is considered an important construct that moderates reactions to stressful situations and directly affects optimism and happiness [24,28,41]. In this sense, self-compassion makes people more joyful and sociable, so that they experience positive feelings, which are considered a fundamental component of happiness. Since our study focused on the restricted times of the pandemic, it is interesting to note that our results are, also, consistent with the recent work of Matos et al. [32], which demonstrate that the characteristics and qualities of people with self-compassion acted as a moderator, protecting them from negative symptoms and stressful feelings caused by the threat of the virus. De Ζoysa et al. [33] also argue that self-compassion is the basis for subjective happiness during a pandemic.

The strong relationship between self-compassion and psychological well-being has been established in a number of studies [17,18,19,34,35,41,42]. Self-compassion has also been shown to be a key factor in psychological well-being during the pandemic, confirming our findings, as it provides people with a sense of safety and security and helps them to combat major negative emotions [43]. Moreover, self-compassion not only has a strong effect on psychological well-being, but also proves to be a very effective weapon against the distress caused by the virus and subsequently mitigates the negative psychological consequences [44]. Based on these findings, we strongly believe that self-compassionate people do live in a sense of psychological wellness.

Furthermore, our study indicated that resilience fully mediated the relationship between self-compassion and psychological well-being with a significant indirect effect and a direct effect. This finding can be attributed to the fact that people with high levels of self-compassion levels tend to be more lenient with themselves and are not stressed or burned out in the face of obstacles and difficulties in life. According to a recent work by Anli and Bilgin [45], developing subjective happiness, resilience, and putting aside one’s sense of self to increase their sense of belonging, are critical to maintaining a positive attitude when it comes to the fear of the COVID-19 pandemic. That is, it appears that resilient and self-transcendent people are happier and better protected from the fear of the virus, which supports our findings.

In addition, the results indicated a positive relationship between self-compassion and meaning in life, such that self-compassion may lead to a more meaningful life. Work by Voetter and Schnell [46] on a population of highly intelligent adults highlighted that developing self-compassion can lead to subjective well-being and meaning in life, as confirmed by previous studies [47]. The direct relationship between self-compassion and resilience and meaning in life was underscored in a recent study by Chan et al. [48], which indicated that self-compassionate students were more resilient while experiencing lower levels of stress and higher levels of meaning in life. These same findings about the significant role of self-compassion and the need to be perfect in providing a deeper sense of meaning in life were confirmed in the work of Suh and Chong [49]. This strong relationship between self-compassion and meaning in life becomes even more important when it comes to the stressful times of the COVID-19 pandemic, as it seems that having a good time while being kind to oneself definitely contributes to having a sense of purpose in life and protects from the negative effects of the pandemic [50,51].

Finally, survey participants in our study who indicated high levels of meaning in life had high levels of subjective happiness, and showed higher levels of psychological well-being. Also, subjective happiness mediated the relationship between self-compassion and psychological well-being. On the other hand, subjective happiness mediated the relationship between resilience and psychological well-being with significant indirect and direct effects.

Consequently, self-compassion, resilience, psychological well-being, subjective happiness, and meaning in life were all intra-related in the study sample. These variables appear to form a unit or group of positive forces that can support each other and act as buffers against life stressors, such as the COVID-19 disease and quarantine. These findings also suggest that self-compassion is a prerequisite for resilience, which in turn may serve as a moderator of psychological well-being and subjective happiness.

## 5. Conclusions

The subject of psychological well-being and mental health is of paramount importance in the science of psychology nowadays. This importance is further emphasized by the critical and stressful times we are experiencing due to the COVID-19 pandemic. The harder and more challenging our lives become, the greater the need for all of humanity to psychologically endure all adversity. Psychologists should address this need by proposing realistic, pragmatic, and applicable models for improving psychological health and well-being. A holistic approach to the happiness prescription could or should incorporate as many as possible of the important new-age variables that come from the field of positive psychology, such as mindfulness, optimism, companionship, positive emotions, the upward spiral proposed by Fredrickson and Losada [52], humor, hope, and spirituality to name a few. These could be inserted into a complex, advanced model of psychological well-being that could help psychology advance in the new era of results-based scientific approach.

As with all research, the present paper has a few limitations worth considering. First, all data were collected through self-report tests, which leaves a wide margin for subjective judgment and understanding by the participants. A more advanced research design utilizing more concrete and objective methods of data collection through interviews and observation should be preferred in the future. Second, the sample was not randomly selected but was approached via email using the snowball technique. Although most results were statistically significant, a larger and more representative sample would be useful for future follow-up research on the same variables. In addition, some subcategories of the sample were underrepresented, while others were overrepresented, leading to problems regarding the balance of the sample. Finally, despite the advanced statistical models and algorithms used in the results, the study design remains in reality survey research and strictly correlational. An interesting approach could have included a more advanced experimental or randomized trial methodology using the same variables.

## Figures and Tables

**Table 1:** Sample demographics.

		No.	%
Gender	Male	51	9.70%
	Female	475	90.30%
Agegroups	22–30	222	42.21%
	31–38	133	25.29%
	39–48	124	23.57%
	49–56	37	7.03%
	57–61	10	1.90%
Education	High school	8	1.52%
	Bachelor’sdegree	340	64.64%
	Master’sdegree	147	27.95%
	PhD	31	5.89%
Marital status	Single	278	52.85%
	Married	233	44.30%
	Divorced/Separated	15	2.85%
Student	No	100	19.00%
	Yes	426	81.00%
Employment	Unemployed	93	17.7%
	Yes, in the tertiary education	44	8.4%
	Yes, outside the tertiary education	389	74%

**Table 2:** Reliability analysis of the tools of the study.

Scales	Cronbach’s Alpha
Subjective happiness scale (SHS)	0.825
DESMOD negative emotions 8 items	0.811
DESMOD positive emotions 9 items	0.882
The Connor–Davidson resilience scale (CD-RISC) 25 items	0.919
Self-compassion scale (SCS) 26 items	0.925

**Table 3:** Pearson correlation coefficients between the scale variables.

	1	2	3	4	5	6
Life satisfaction	--					
Self-compassion	0.447 **	--				
Subjective happiness	0.656 **	0.607 **	--			
Meaning of life	0.384 **	0.382 **	0.410 **	--		
Resilience	0.541 **	0.610 **	0.591 **	0.555 **	--	
Psychological well-being	0.493 **	0.569 **	0.625 **	0.321 **	0.500 **	--

** *p* < 0.01.

**Table 4:** Path coefficients and indirect effects for mediation of resilience in the relationship between self-compassion and psychological well-being.

	Path Coefficients		Indirect Effects	
	To Resilience	To Psychological Well-Being	Estimate	95%CI
Self-compassion	0.507(0.030)	0.024(0.003)		
Resilience		0.016(0.003)		
Total			0.032(0.002)	0.028, 0.036
Indirect (SC→RES→WB)			0.008(0.002)	0.005, 0.011
Direct (SC→WB)			0.023(0.003)	0.019, 0.028

**Table 5:** Path coefficients and indirect effects for mediation of subjective happiness in the relationship between self-compassion, meaning of life, and resilience with psychological well-being.

	Path Coefficients		Indirect Effects	
	To Subjective Happiness	To Psychological Well-Being	Estimate	95%CI
Self-compassion	0.109(0.011)			
Subjective happiness		0.081(0.009)		
Meaning of life	0.048(0.020)			
Resilience	0.102(0.015)			
Total (SC→WB)			0.023(0.003)	0.018, 0.029
Indirect (SC→SH→WB)			0.009(0.001)	0.001, 0.007
Direct (SC→WB)			0.015(0.003)	0.009, 0.019
Total (ML→WB)			0.004(0.004)	−0.004, 0.012
Indirect (ML→SH→WB)			0.004(0.002)	0.005, 0.011
Direct (ML→WB)			−0.002(0.004)	−0.008, 0.008
Total (RES→WB)			0.015(0.003)	0.009, 0.022
Indirect (RES→SH→WB)			0.008(0.001)	0.005, 0.011
Direct (RES→WB)			0.007(0.003)	0.001, 0.013

**Table 6:** Hierarchical linear regression results for the effect of self-compassion on subjective happiness.

		Unstandardized			
		B	SE	β	*p*
Model 1	(Constant)	5.313	0.825		<0.001
	Self-compassion	0.169	0.010	0.603	<0.001
Model 2	(Constant)	1.765	1.009		0.081
	Self-compassion	0.147	0.010	0.522	<0.001
	Meaning in life	0.107	0.019	0.212	<0.001
Model 3	(Constant)	0.562	0.985		0.569
	Self-compassion	0.106	0.012	0.378	<0.001
	Meaning in life	0.049	0.020	0.097	0.013
	Resilience	0.104	0.015	0.308	<0.001
Model 4	(Constant)	1.054	1.09		0.334
	Self-compassion	0.11	0.012	0.391	<0.001
	Meaning in life	0.053	0.02	0.104	0.008
	Resilience	0.096	0.016	0.286	<0.001
	Gender (Male vs. Female)	−0.975	0.536	−0.061	0.07
	Age	−0.336	0.201	−0.075	0.095
	Has PhD	0.222	0.897	0.011	0.804
	Has master’s degree	−0.07	0.348	−0.007	0.841
	Is married	0.09	0.486	0.009	0.854
	Is divorced/separated	−1.328	1.038	−0.047	0.201
	Number of children	0.52	0.242	0.111	0.032
	Is a student	0.012	0.445	0.001	0.978
	Is unemployed	−0.428	0.417	−0.034	0.305
	Is employed in tertiaryeducation	0.361	0.722	0.021	0.617

Note: Model 1. *F*(1, 519) = 296.97, *p <* 0.001, adjusted *R^2^* = 0.363, model 2. *F*(2, 518) = 174.45, *p <* 0.001, adjusted *R*^2^ = 0.400, model 3. *F*(3, 517) = 141.29, *p <* 0.001, adjusted *R*^2^ = 0.451, model 4. *F*(13, 507) = 34.27, *p <* 0.001, adjusted *R*^2^ = 0.468.

**Table 7:** Hierarchical linear regression results for the effect of subjective happiness on psychological well-being.

		Unstandardized			
		B	SE	β	*p*
Model 1	(Constant)	−0.254	0.135		0.061
	Subjective happiness	0.124	0.007	0.625	<0.001
Model 2	(Constant)	−1.074	0.196		<0.001
	Subjective happiness	0.082	0.009	0.413	<0.001
	Self-compassion	0.015	0.002	0.261	<0.001
	Meaning in life	−0.001	0.004	−0.008	0.846
	Resilience	0.007	0.003	0.097	0.042
Model 3	(Constant)	−1.016	0.22		<0.001
	Subjective happiness	0.082	0.009	0.41	<0.001
	Self-compassion	0.015	0.003	0.265	<0.001
	Meaning in life	0	0.004	−0.004	0.915
	Resilience	0.007	0.003	0.1	0.04
	Gender (Male vs. Female)	−0.056	0.109	−0.017	0.608
	Age	0.004	0.041	0.005	0.92
	Has PhD	−0.091	0.181	−0.023	0.615
	Has master’s degree	−0.111	0.07	−0.053	0.116
	Is married	0.002	0.098	0.001	0.983
	Is divorced/separated	−0.04	0.21	−0.007	0.848
	Number of children	−0.02	0.049	−0.021	0.684
	Is a student	−0.046	0.09	−0.019	0.608
	Is unemployed	−0.04	0.084	−0.016	0.635
	Is employed in tertiary education	−0.005	0.146	−0.001	0.973

Note: Model 1. *F*(1, 519) = 331.98, *p* < 0.001, adjusted *R*^2^ = 0.390, model 2. *F*(4, 516) = 106.21, *p* < 0.001, adjusted *R*^2^ = 0.452, model 3. *F*(14, 506) = 30.24, *p* < 0.001, adjusted *R*^2^ = 0.456.

**Table 8:** Hierarchical linear regression results for the effect of psychological well-being on subjective happiness.

		Unstandardized			
		B	SE	β	*p*
Model 1	(Constant)	12.537	0.402		<0.001
	Psychological well-being	3.136	0.172	0.625	<0.001
Model 2	(Constant)	2.384	0.932		0.011
	Psychological well-being	1.773	0.189	0.353	<0.001
	Self-compassion	0.065	0.012	0.231	<0.001
	Meaning in life	0.043	0.018	0.086	0.018
	Resilience	0.077	0.015	0.229	<0.001
Model 3	(Constant)	2.662	1.026		0.01
	Psychological well-being	1.729	0.19	0.344	<0.001
	Self-compassion	0.069	0.012	0.244	<0.001
	Meaning in life	0.046	0.018	0.091	0.013
	Resilience	0.071	0.015	0.211	<0.001
	Gender (Male vs. Female)	−0.741	0.498	−0.046	0.138
	Age	−0.296	0.186	−0.066	0.113
	Has PhD	0.348	0.833	0.017	0.676
	Has master’s degree	0.131	0.324	0.012	0.686
	Is married	0.073	0.451	0.008	0.871
	Is divorced/separated	−1.071	0.964	−0.038	0.267
	Number of children	0.481	0.225	0.102	0.033
	Is a student	0.09	0.413	0.008	0.827
	Is unemployed	−0.299	0.387	−0.024	0.441
	Is employed in tertiary education	0.319	0.67	0.019	0.634

Note: Model 1. *F*(1, 519) = 331.98, *p* < 0.001, adjusted *R*^2^ = 0.390, model 2. *F*(4, 516) = 145.81, *p* < 0.001, adjusted *R*^2^ = 0.531, model 3. *F*(14, 506) = 42.92, *p* < 0.001, adjusted *R*^2^ = 0.543.

## Data Availability

Not applicable.
